# Palmitoylethanolamide for sleep disturbance. A double-blind, randomised, placebo-controlled interventional study

**DOI:** 10.1186/s41606-021-00065-3

**Published:** 2021-09-10

**Authors:** Amanda Rao, Phillippa Ebelt, Alistair Mallard, David Briskey

**Affiliations:** 1 RDC Clinical, Brisbane, 4006 Australia; 2 University of Sydney, School of Medicine, Sydney, Australia; 3 Faculty of Medicine, University of Queensland, Brisbane, Australia; 4 School of Human Movement and Nutrition Sciences, University of Queensland, Brisbane, Australia

**Keywords:** Palmitoylethanolamide, Levagen, Sleep, Sleep onset

## Abstract

**Background:**

Sleep is essential for wellbeing, yet sleep disturbance is a common problem linked to a wide range of health conditions. Palmitoylethanolamide (PEA) is an endogenous fatty acid amide proposed to promote better sleep via potential interaction with the endocannabinoid system.

**Methods:**

This double-blind, randomised study on 103 adults compared the efficacy and tolerability of 8 weeks of daily supplemented PEA formulation (350 mg Levagen + ®) to a placebo. Sleep quality and quantity were measured using wrist actigraphy, a sleep diary and questionnaires.

**Results:**

At week 8, PEA supplementation reduced sleep onset latency, time to feel completely awake and improved cognition on waking. After 8 weeks, both groups improved their sleep quality and quantity scores similarly. There was no difference between groups at baseline or week 8 for sleep quantity or quality as measured from actigraphy or sleep diaries.

**Conclusion:**

These findings support PEA as a potential sleeping aid capable of reducing sleep onset time and improving cognition on waking.

**Trial registration:**

Australian New Zealand Clinical Trials Registry ACTRN12618001339246. Registered 9^th^ August 2018.

## Introduction

Sleep is essential for mental and physical wellbeing (Chattu et al. 2018; Medic et al. 2017). Having a properly functioning sleep–wake cycle promotes survival (Worley 2018), adequate energy levels and normal motor and cognitive functioning (Walker et al. 2003; Kesner and Lovinger 2020). Impaired sleep has been linked to a number of health consequences (Medic et al. 2017) such as: negative social functioning, accidents, cardiovascular disease, chronic pain, neurodegenerative disorders, depression, obesity, cancer and all-cause mortality (Chattu et al. 2018; Medic et al. 2017; Irwin et al. 2015; Krueger et al. 2008; Mullington et al. 2010). An underlying pathogenesis to these health conditions is inflammation. Poor sleep quality has been shown to increase inflammatory mediators, potentially inducing the adverse physical and cognitive symptoms of sleep loss (Irwin et al. 2015, 2016; Krueger et al. 2008; Mullington et al. 2010). Poor sleep quality may also increase inflammation through increased sympathoadrenal activity, decreased glucose tolerance and neuroendocrine changes (Irwin et al. 2015; Krueger et al. 2008; Mullington et al. 2010). Therefore, sleep disruption and increased inflammation could become a bidirectional relationship.

The endogenous cannabinoid (endocannabinoid) system regulates numerous circadian processes including food intake, peripheral metabolism, and body temperature via the suprachiasmatic nucleus (Vaughn et al. 2010; Ho et al. 2008; Prospero-Garcia et al. 2016; Murillo-Rodriguez et al. 2011) and is involved in the sleep/wake cycle, (Kesner and Lovinger 2020; Murillo-Rodriguez et al. 2011). The endocannabinoid system consists of lipid mediators that act upon specific receptors, including the nervous system (Kesner and Lovinger 2020; Prospero-Garcia et al. 2016; Murillo-Rodriguez et al. 2011). It can also influence temperature regulation, fat storage, mood and behaviour regulation, sensory perception, motor activity, nervous system modulation, and endocrine and gastrointestinal (GI) function (Vaughn et al., 2010) – all previously shown to have an effect on sleep (Vaughn et al., 2010). Endocannabinoid signalling follows a circadian rhythm (Vaughn et al. 2010; Murillo-Rodriguez et al. 2011), such that sleep deprivation could lead to disruption to this cycle (Vaughn et al., 2010). Developing treatment strategies that target the endocannabinoid system could be a potential way to manage sleep disturbances. It is important to note that sleep deprivation and sleep disturbance are different. In the context of this study, sleep deprivation is defined as inadequate quantity or quality of sleep, whereas sleep disturbance, as used in this study, is generally difficulty getting to sleep and/or an inability to maintain sleep throughout the night.

Palmitoylethanolamide (PEA), an endogenous fatty acid amide, works synergistically with the endocannabinoid, anandamide (AEA) (Ho et al. 2008). AEA concentrations are low at sleep onset, increase during sleep and high at wakening (Vaughn et al. 2010; Ho et al. 2008). It is proposed that increased AEA signalling could facilitate deep non-rapid eye movement (NREM) sleep through inducing adenosine release (Prospero-Garcia et al. 2016). However, disturbed sleep is possibly related to impaired AEA signalling (Vaughn et al. 2010). Therefore, an exogenous dose of PEA could possibly restore dysregulated AEA signalling and facilitate better sleep. PEA is also proposed to have an effect on sleep due to its ability to act through transient receptor potential cation channel subfamily V member 1 (TRPV1). Activation of TRPV1 via increased AEA initiates vasorelaxation through a release of vasodilators (Vaughn et al. 2010; Zygmunt et al. 1999) and may facilitate sleep (Vaughn et al. 2010; Ho et al. 2008; Ambrosino et al. 2013; Franco-Cereceda and Rudehill 1989).

With sleep deprivation shown to increase pain sensitivity (Staffe et al. 2019) and the link between pain and sleep quality documented (Finan et al. 2013; Gerhart et al. 2017), finding ways to reduce pain may help increase sleep quality. The anti-inflammatory and other immune-modulating properties of PEA have been shown in several placebo-controlled double-blind clinical studies (Keppel Hesselink et al. 2013; Guida et al. 2017; Heide et al. 2018). PEA is thought to exert an antagonistic action against inflammation and pain receptor stimulation by down regulating mast cell degranulation at local sites (Re et al. 2007; Gatti et al. 2012; Mattace Raso et al. 2014; Skaper et al. 2015; Seol et al. 2017). Additionally, PEA’s pain-alleviating and anti-inflammatory properties (Canteri et al. 2010; Evangelista et al. 2018; Chirchiglia et al. 2018; Conigliaro et al. 2011; Dalla Volta et al. 2016; Keppel Hesselink and Hekker 2012; Marini et al. 2012) could reduce pain and inflammation reported to impair sleep (Evangelista et al. 2018). A study by Evangelista et al. found that 600 mg of PEA administered to patients awaiting carpal tunnel syndrome surgery significantly improved patient’s overall sleep quality, including an increase in continuous sleep time and a reduction of sleep latency (Evangelista et al. 2018). It was reported that this was due to its mitigation of pain symptoms in the treatment group encountering neuropathic pain, which contributed to their poor sleep quality (Evangelista et al. 2018). Such findings point to PEA as a potential option for sleep disturbance and warrants further investigation.

A limitation to PEA’s therapeutic efficacy is its traditionally poor bioavailability (Gabrielsson et al. 2016). Levagen + ® is a clinically studied PEA formulation utilizing cold-water dispersible (CWD) technology (LipiSperse®) that significantly increases plasma PEA concentrations by approximately twofold (Briskey et al. 2020). Therefore, the aim of this study is to evaluate the efficacy of Levagen + ® (PEA) supplementation on sleep quality and quantity in healthy adults with sleep pattern disturbance. It is hypothesised that PEA supplementation one hour prior to sleep onset will improve sleep quality, quantity and onset time.

## Materials and methods

This study was approved by the Bellberry Ltd Human research and ethics committee (approval number HREC2018-08–668-A-1) and carried out in accordance with current International Conference on Harmonization Guideline for Good Clinical Practice and registered on the Australian New Zealand Clinical Trials Registry (ACTRN12618001339246). Potential participants were recruited through databases and mainstream media.

### Participants

One hundred and twenty-five healthy males and females over 18 years of age with a disturbed sleeping pattern [> 5 on the Pittsburgh Sleep Quality Index (PSQI)] were recruited from across Australia to participate in this at home study. Potential participants were screened for inclusion and exclusion criteria prior to providing written consent for enrolment. Inclusion criteria included: males and females, females of child-bearing potential were required to be on a prescribed form of birth control, agree not to change current diet or exercise or use other supplements for sleep disturbances for the study period. Exclusion criteria included: any unstable or serious illness (e.g. kidney, liver, diabetes), malignancy or treatment for malignancy within the previous two years, clinically significant inflammation connective tissue disease or arthritis, suffered from any mood disorders such as depression and bipolar disorder, any neurological disorders such as multiple sclerosis, receiving or prescribed over the counter sleep medication or aid during the trial, diagnosed sleep apnea, diagnosed or consistent gastrointestinal issues that disrupt sleep, active smokers or those taking nicotine, prescribed drug or illegal substances, chronic past and/or present alcohol use (> 14 alcoholic drinks per week), regular intake of stimulants (e.g. coffee, caffeine supplements or caffeine containing beverages) from midday onwards, diagnosed with a clinical sleep disorder (e.g. insomnia), night-shift employment or any other such situation that left one unable to have a normal night’s sleep, disturbed sleeping patterns caused by external factors (e.g. children, partner, noise), allergic to any of the ingredients in the PEA or placebo formula, pregnant or lactating or any condition or non-medicated supplement use which in the opinion of the investigator made the participant unsuitable for inclusion.

Participants were randomly allocated to either the placebo or PEA group using random allocation software (www.sealedenvelope.com), with both the participants and investigators blinded to allocation.

Participants received either 350 mg of PEA (2 × 175 mg Levagen + ® capsules) or maltodextrin (2 × 175 mg capsules) taken orally, daily, and with water one hour prior to sleep for the study duration (8 weeks). The PEA and placebo product were both housed in opaque capsules and bottles to appear identical. Trial product was supplied by Gencor Pacific (Lantau Island, Hong Kong) and manufactured by Pharmako Biotechnologies (Sydney, Australia).

### Intervention

Upon enrolment, participants completed a health assessment including diet, exercise, medication and medical history, anthropometry measures (weight, height, body mass index, waist/hip circumference), questionnaires PSQI, The Sleep Inertia Questionnaire (SIQ), Epworth Sleepiness Scale (ESS), Patient Reported Outcomes Measurement Information System (PROMIS) sleep disturbance, Rand 36-item Health Survey (SF-36) and gastrointestinal tolerance questionnaire] and provided a blood sample (when possible).

Upon completion of baseline measures, participants were randomised and provided with trial product as well a Consensus Sleep Diary (Carney et al. 2012) and Polar (Kempele, Finland) A370 sleep monitor both of which were completed/worn for 3 consecutive days/nights at baseline, day 5 and weeks 2, 4, and 8. During the trial, questionnaires were repeated on day 5 and weeks 2 and 4. Upon completion (week 8), all baseline measures were again recorded. (Table 1).

**Table 1:** Outcome Measures

Outcome measure	Frequency	Tool used
Sleep Quality and Quantity	Baseline, day 5, weeks 2, 4 & 8	PSQI, Polar A370 sleep monitor (wrist actigraphy) and the consensus sleep diary
Sleep onset latency	Baseline, day 5, weeks 2, 4 & 8	Consensus sleep diary
Sleep disturbance (waking during the night or waking too early)	Baseline, day 5, weeks 2, 4 & 8	Wrist actigraphy, consensus sleep diary and the PROMIS sleep disturbance questionnaire
Sleep inertia	Baseline, day 5, weeks 2, 4 & 8	SIQ and wrist actigraphy
Daytime sleepiness	Baseline, day 5, weeks 2, 4 & 8	SIQ and consensus sleep diary
Morning grogginess on waking	Baseline, day 5, weeks 2, 4 & 8	SIQ and consensus sleep diary
Daytime nap count and duration	Baseline, day 5, weeks 2, 4 & 8	ESS and consensus sleep diary
General quality of life	Baseline, day 5, weeks 2, 4 & 8	SF-36
Pathology and Safety markers	Baseline and week 8	Albumin, ALT, AST, GGT, total bilirubin, hsCRP, IL10, IL6, IL8 and TNFa
Adverse events	Continuously	Spontaneously reported by the participant or noticed by a trial supervisor

*PSQI* Global Pittsburgh Sleep Quality Index, *PROMIS* Patient Reported Outcomes Measurement Information System, *SIQ* The Sleep Inertia Questionnaire, *ESS* Epworth Sleepiness Scale, *SF-36* Rand 36-item Health Survey, *ALT* Alanintransaminase, *AST* Aspartate Transaminase, *GGT* Gamma-Glutamyl Transferase, *hsCRP* high sensitivity C-reactive Protein, *IL* interleukin, *TNF* Tumor Necrosis Factor

### Statistical analysis

A sample size of 100 participants was required, based on the power to detect a change of 2 PSQI points between the placebo and treatment groups using and effect size: 0.67, Alpha error probability: 0.05, Power 0.95. Based on an approximate 20% drop-out rate 125 participants were recruited.

Data was analysed with R (Vienna, Austria), using a range of native statistical functions and functions from the packages tidyverse, rcompanion, dplyr, reshape2, and ggplot. Linear regression slope calculation for later analysis was performed in Microsoft Excel. Once assessed for normality, differences between group means were assessed with both Student’s t-test and Mann–Whitney U (non-parametric) tests, accordingly. Linear modelling via repeated measures ANOVA was carried out to test further the likelihood of a significant difference between products over the length of the study. Repeated measures ANOVA/ANCOVA were used to analyze within groups variables from all time points, with and without covariates. General linear mixed modelling was also performed on participant data to compare the group dynamics. Results were considered statistically significant if *p* < 0.05.

Analysis for sleep onset latency was undertaken only for participants where sleep latency was more than 10 min at baseline (n = 78 participants).

## Results

Of the 125 randomized participants, 103 completed the study (55 PEA and 48 placebo), 16 were lost to follow up and six dropped out due to adverse events: two in the PEA group (loss of taste and reduced sleep quality) and four in the placebo group (stomach pain, diarrhoea, dizziness, worse sleep).

No significant differences were observed between the two groups for any baseline demographic data. (Table 2).

**Table 2:** Baseline demographic results

	**PEA**	**Placebo**
Male	25	14
Female	30	34
Waist circumference (cm)	91.6 ± 14.4	89.4 ± 14.0
Hip circumference (cm)	104.6 ± 10.4	104.0 ± 7.9
Heart rate (bpm)	63.2 ± 9.3	62.9 ± 9.3
Height (cm)	171.3 ± 8.2	170.9 ± 10.9
Weight (kg)	79.4 ± 17.6	75.7 ± 17.1

N.B. Data is presented as mean ± standard deviation; *PEA* palmitoylethanolamide, *bpm* beats per minute

Sleep quality was rated as moderate for both groups from the PSQI (Buysse et al. 1989) score with no difference between groups at baseline present (Table 3). At week 8, both groups had improved their PSQI scores similarly (Table 3).

**Table 3:** The Pittsburgh Sleep Quality Index

	**PEA**	**Placebo**
Baseline	11.2 ± 2.8	11.7 ± 3.3
Day 5	9.0 ± 2.7	9.8 ± 3.4
Week 2	8.2 ± 2.7	8.8 ± 3.3
Week 4	7.8 ± 3.0	8.2 ± 3.4
Week 8	7.6 ± 3.1	7.7 ± 3.6

N.B. Data presented as mean ± standard deviation; *PEA* palmitoylethanolamide

**Fig. 1:**
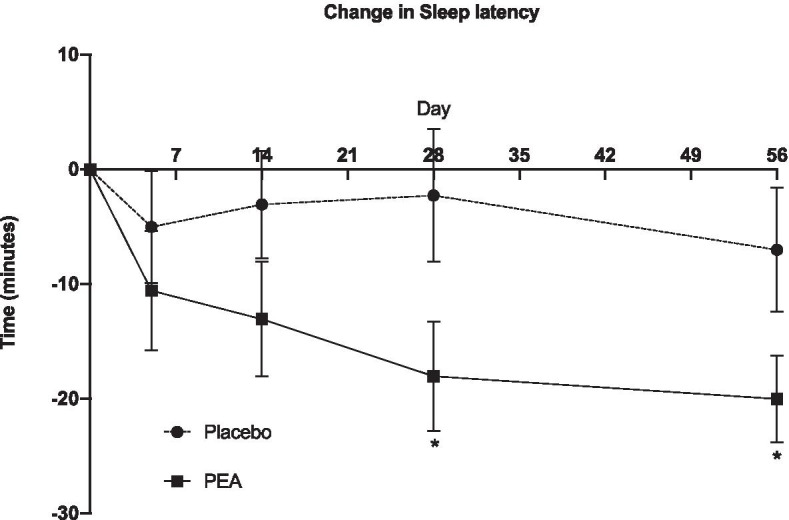
Change in Sleep onset latency (time to get to sleep) over 8 weeks for participants reporting time to sleep > 10 min at baseline

There was no significant difference in sleep quantity or quality for actigraphy measurements or sleep diaries at baseline or 8 weeks between groups (Tables 4 and 5). There was no significant difference for sleep onset latency (time to fall asleep) at baseline between groups (Table 5). A sub-group analysis utilising general linear mixed model analysis for individuals with sleep onset > 10 min at baseline showed a significant reduction in sleep onset latency time (total and change) for weeks 4 and 8 in the PEA group compared with the placebo group (Table 4; Fig. 1; *p* < 0.05).

**Table 4:** Sleep actigraphy data

	**PEA**			**Placebo**		
	**Baseline**	**Week 4**	**Week 8**	**Baseline**	**Week 4**	**Week 8**
Total Sleep time (hrs)	7.49 ± 1.1	7.66 ± 1.2	7.58 ± 1.1	7.52 ± 1.2	7.54 ± 1.2	7.48 ± 1.1
Sleep Time (hrs)	7.00 ± 1.0	7.10 ± 1.0	7.03 ± 1.1	6.98 ± 1.2	7.02 ± 1.2	6.97 ± 1.1
Interruptions (min)	34.3 ± 14.1	32.3 ± 11.6	32.2 ± 11.2	32.1 ± 12.2	31.3 ± 12.4	30.9 ± 11.8
Sleep quality (1–5)	2.7 ± 0.6	3.1 ± 0.7	3.1 ± 0.7	2.5 ± 0.7	3.0 ± 0.7	2.9 ± 0.9
Sleep Continuity (1–5)	3.2 ± 0.9	3.2 ± 0.9	3.2 ± 0.9	3.2 ± 0.9	3.4 ± 1.0	3.3 ± 0.9
Sleep Percentage (%)	92.4 ± 3.9	92.9 ± 3.0	92.8 ± 2.4	92.8 ± 3.0	91.1 ± 7.5	93.0 ± 2.9

N.B. Data presented as mean ± standard deviation; total sleep time = sleep time + interruptions + sleep latency; *PEA* palmitoylethanolamide

**Table 5:** Sleep diary data

	**PEA**			**Placebo**		
	**Baseline**	**Week 4**	**Week 8**	**Baseline**	**Week 4**	**Week 8**
Sleep time (hrs)	6.1 ± 1.5	6.7 ± 1.4	6.7 ± 1.3	6.1 ± 1.5	6.5 ± 1.3	6.5 ± 1.5
Sleep Latency^a^ (min)	42.8 ± 42.2	26.8 ± 26.3^*^	23.4 ± 23.1^*^	36.2 ± 40.5	34.2 ± 40.5	30.3 ± 39.2
Interruptions (n)	3.3 ± 2.1	3.0 ± 2.3	3.3 ± 3.0	3.9 ± 3.8	3.0 ± 3.0	2.9 ± 2.8
Interruptions (min)	41.0 ± 39.3	29.9 ± 30.1	35.0 ± 34.8	35.3 ± 32.0	31.6 ± 41.5	32.6 ± 42.7

N.B. Data presented as mean ± standard deviation; * = significant difference between groups; ^a^ sleep latency data for participants reporting > 10 min at baseline (n = 78); *PEA* Palmitoylethanolamide

There was no change in sleep disturbance or sleep interruptions as measured by consensus sleep diary, actigraphy or the PROMIS questionnaire during the study (Tables 4, 5 and 6). There were no differences in daytime sleepiness levels (ESS) for both groups throughout the study (Table 6).

**Table 6:** Data from sleep questionnaires including domain scores

	**PEA**			**Placebo**		
	**Baseline**	**Week 4**	**Week 8**	**Baseline**	**Week 4**	**Week 8**
Epworth Sleepiness Scale	6.5 ± 4.4	4.6 ± 3.8	4.7 ± 4.1	6.7 ± 4.1	4.8 ± 3.9	4.3 ± 4.1
PROMIS	61.5 ± 6.0	56.8 ± 6.5	55.3 ± 6.6	62.2 ± 6.5	56.5 ± 6.7	55.1 ± 9.0
SIQ Total score	45.1 ± 15.7	36.9 ± 13.1	37.1 ± 12.4	45.0 ± 12.4	37.1 ± 15.0	36.8 ± 15.1
SIQ Physiological	15.8 ± 6.0	12.2 ± 4.5	12.4 ± 4.7	16.2 ± 4.9	12.8 ± 5.7	12.6 ± 5.5
SIQ Responses to Sleep Inertia	13.8 ± 5.2	12.3 ± 4.9	11.6 ± 4.3	14.5 ± 4.6	12.0 ± 4.7	12.0 ± 5.0
SIQ Cognitive	10.5 ± 4.6	9.0 ± 3.7	8.0 ± 3.2^a^	9.4 ± 3.0	7.4 ± 3.6	7.8 ± 4.1
SIQ Emotional	4.9 ± 2.6	4.4 ± 2.3	4.2 ± 1.6	4.9 ± 2.3	4.5 ± 2.7	4.8 ± 2.8
SIQ Time to feel completely awake	25.9 ± 25.3	18.4 ± 21.1	14.7 ± 17.5^a^	23.6 ± 23.8	20.4 ± 20.4	22.4 ± 25.1

N.B. Data presented as mean ± standard deviation;  ^a^significant difference between groups at *p* < 0.05; *PEA*  Palmitoylethanolamide, *PROMIS* Patient Reported Outcomes Measurement Information System, *SIQ* The Sleep Inertia Questionnaire

There was a significant difference (*p* < 0.05) in change from baseline for time to feel completely awake (sleep inertia) and domain scores for cognition in the PEA group compared to the placebo at week 8. No other domains were significantly different between groups (Table 6).

No significant differences either within or between groups were found for any pathology marker or for the SF-36. All pathology markers were within normal ranges throughout the study. There was no difference between the placebo and PEA group for compliance (9.7 ± 8.4 and 13.2 ± 9.9 capsules remaining respectively) or reported gastrointestinal tolerance issues between groups (4 reported cases in the placebo group).

## Discussion

The aim of this study was to evaluate the efficacy of PEA supplementation on sleep quality and quantity in healthy adults with sleep pattern disturbance. It was hypothesised that PEA supplementation one hour prior to sleep would improve sleep quality, quantity and onset time. No treatment effect was seen for the total PSQI score or sleep duration, despite both groups reporting poor sleep at baseline as measured by the PSQI. This is due to both groups reporting a reduction in PSQI scores from baseline scores.

The reduction in PSQI in both groups could be due to a number of possible reasons. One reason may be a placebo effect. A second possibility is that participants may have become more focussed on measuring individual aspects their sleep. As such, baseline scores may be inflated, with participants not having an accurate reflection of their sleep behaviour. However, after 8-weeks in the study, participants may have had an improved recall on the facets of their sleep such that the PSQI total score may have increased in accuracy.

To accurately measure sleep duration, two methods of sleep measurement were incorporated in the study. Sleep duration was measured by both consensus sleep diary and wrist actigraphy which were consistent between measures but overall, slightly lower, though not significant, in consensus diary reporting (0.3–0.8 h). Previous research has demonstrated a moderate correlation between diary and actigraphy measures, with discrepancies higher in groups who have less sleep (Carney et al. 2012; Hanish et al. 2017). In our study, both groups reported achieving approximately 6–7 h of sleep per night. Therefore, it is likely that the study population recruited in our study suffered more from sleep disturbance and high sleep onset latency rather than lack of sleep duration. This is likely due to the inclusion criteria set to primarily recruit those reporting sleep disturbance rather than sleep disorders, with anyone previously diagnosed with a sleep disorder, such as insomnia, excluded from participating.

A key finding of this study indicated that PEA significantly reduced the amount of time to fall asleep in individuals with sleep latency issues. This reduction in sleep onset latency could be the result of a number of physiological responses to PEA. An increase in AEA levels via the endocannabinoid system (Vaughn et al. 2010; Ho et al. 2008), a change in inflammatory signalling or reduction in pain sensitivity could all promote faster sleep (Betoni et al. 2013; Di Cesare et al. 2013; Helyes et al. 2003; Costa et al. 2008; LoVerme et al. 2006; Luongo et al. 2013). Sleep disturbance has been linked with inflammation and inflammatory signalling that contributes to possible sleep disturbance (Irwin et al. 2015; Mullington et al. 2010; Irwin et al. 2016). Therefore, a change in inflammation sensitivity (signalling or receptor activity) could alter sleep patterns. However, as this trial did not measure AEA concentrations, or find any change in serum cytokines, this is speculative.

PEA supplementation improved the time to feel fully awake and cognition on waking. This is of particular interest as sleep inertia and daytime grogginess is a common side effect of many pharmaceutical options for the treatment of sleep disturbance (Miner and Kryger 2017). The combination of the PEA group reporting falling asleep faster and waking up feeling more alert and awake compared to the placebo group, suggests that future studies on PEA and sleep should focus on populations with difficulty getting to sleep and/or waking up.

A major limitation of this study was that it was disrupted by the COVID-19 pandemic, limiting the number of participants able to provide blood samples. Therefore, we were only able to measure pathology markers on a limited number of participants greatly reducing the power of the analysis making it difficult to make inferences on the outcomes. Had all participants been able to provide a blood sample, it may have provided stronger evidence for changes in blood cytokines and supported an anti-inflammatory mechanism. A proposed area of focus for future studies on PEA and sleep would benefit by having a studied powered for biochemistry markers such as AEA and inflammatory cytokines.

## Conclusion

Overall, the results of this study support PEA as a potential sleeping aid capable of reducing sleep onset time in individuals with sleep latency issues and improving cognition on waking. Further studies would benefit by specifically focusing on sleep latency and/or including participants with severely disturbed sleep.

## Data Availability

The datasets generated and/or analysed during the current study are not publicly available due to commercial agreements, but are available from the corresponding author on reasonable request.

## Abbreviations

PEA: Palmitoylethanolamide
AEA: Anandamide
NREM: Non-rapid eye movement
TrpV1: Transient receptor potential cation channel subfamily V member 1
CWD: Cold-water dispersible
PSQI: Global Pittsburgh Sleep Quality Index
SIQ: The Sleep Inertia Questionnaire
ESS: Epworth Sleepiness Scale
PROMIS: Patient Reported Outcomes Measurement Information System
SF-36: Rand 36-item Health Survey
ALT: Alanintransaminase
AST: Aspartate Transaminase
GGT: Gamma-Glutamyl Transferase
hsCRP: High sensitivity C-reactive Protein
IL: Interleukin
TNF: Tumor Necrosis Factor
BPM: Beats per minute

## References

[CR1] Alhouayek M, Muccioli G (2014). Harnessing the anti-inflammatory potential of palmitoylethanolamide. Drug Discov.

[CR2] Ambrosino P, Soldovieri MV, Russo C (2013). Activation and desensitization of TRPV1 channels in sensory neurons by the PPARalpha agonist palmitoylethanolamide. Br J Pharmacol.

[CR3] Betoni I, Comelli F, Colombo A (2013). Non-neuronal cell modulaion relieves neuropathic pain: eicacy of the endogenous lipid palmitoylethanolamide. CNS Neurol Disord Drug Targets.

[CR4] Briskey D, Mallard AR, Rao A. Increased absorption of palmitoylethanolamide using a novel dispersion system (LipiSperse®). J Nutraceuticals Food Sci. 2020;5:1–6.

[CR5] Buysse DJ, Reynolds CF, Monk TH, Berman SR, Kupfer DJ (1989). The Pittsburgh sleep quality index: a new instrument for psychiatric practice and research. Psychiatry Res.

[CR6] Canteri L, Petrosino S, Guida G (2010). Reduction in the consumption of anti inflammatory and analgesic agents during the treatment of chronic neuropathic pain in patients with compression lumbosciatalgia and treatment with NORMAST ® 300mg. DOLOR.

[CR7] Carney CE, Buysee DJ, Ancoli-Israel S (2012). The consensus sleep diary: standardizing prospective sleep self-monitoring. Sleep.

[CR8] Chattu VK, Manzar MD, Kumary S (2018). The global problem of insufficient sleep and its serious public health implications. Healthcare (Basel)..

[CR9] Chirchiglia D, Cione E, Caroleo MC, Wang M, Di Mizio G, Faedda N, Giacolini T, Siviglia S, Guidetti V, Gallelli L (2018). Effects of add-on ultramicronized n-palmitol ethanol amide in patients suffering of migraine with aura: a pilot study. Front Neruol.

[CR10] Conigliaro R, Drago V, Foster PS, Schievano C, Di Marzo V (2011). Use of palmitoylethanolamide in the entrapment neuropathy of the median in the wrist. Minerva Med.

[CR11] Costa B, Comelli F, Betoni I (2008). The endogenous fatty acid amide, palmitoylethanolamide, has anti-allodynic and ani-hyperalgesic effects in a murine model of neuropathic pain: involvement of CB(1), TRPV1 and PPARgamma receptors and neurotrophic factors. Pain.

[CR12] Dalla Volta G, Zavarize P, Ngonga GK, Carli D (2016). Ultramicronized palmitoylethanolamide reduces frequency and pain intensity in migraine. A pilot study. Int J Neurol Brain Dis.

[CR13] Di CesareMannelli L, D’Agosino G, Pacini A, et al. Palmitoylethanolamide is a disease-modifying agent in peripheral neuropathy: pain relief and neuroprotecion share a PPAR-alpha-mediated mechanism. Mediators Infamm. 2013;2013:328797. 10.1155/2013/328797 PMC3596927 23533304

[CR14] Evangelista MC, De Vitis R (2018). Ultra-micronized palmitoylethanolamide effects on sleep-wake rhythm and neuropathic pain phenotypes in patients with carpal tunnel syndrome: an open-label, randomized controlled study. CNS Neurol Disord Drug Targets.

[CR15] Finan PH, Goodin BR, Smith MT (2013). The association of sleep and pain: an update and a path forward. J Pain.

[CR16] Franco-Cereceda A, Rudehill A (1989). Capsaicin-induced vasodilataion of human coronary arteries in vitro is mediated by calcitonin gene-related peptide rather than substance P or neurokinin A. Acta Physiol Scand.

[CR17] Gabrielsson L, Mattsson S, Fowler C (2016). Palmitoylethanolamide for the treatment of pain: pharmacokinetics, safety and efficacy. Br J Clin Pharmacol.

[CR18] Gatti A, Lazzari M, Gianfelice V (2012). Palmitoylethanolamide in the treatment of chronic pain caused by different etiopathogenesis. Pain Med.

[CR19] Gerhart JI, Burns JW, Post KM (2017). Relationships between sleep quality and pain-related factors for people with chronic low back pain: tests of reciprocal and time of day effects. Ann Behav Med.

[CR20] Guida F, Luongo L, Boccella S (2017). Palmitoylethanolamide induces microglia changes associated with increased migration and phagocytic activity: involvement of the CB2 receptor. Sci Rep.

[CR21] Hanish AE, Lin-Dyken DC, Han JC (2017). PROMIS sleep disturbance and sleep-related impairment in adolescents: examining psychometrics using self-seport and actigraphy. Nurs Res.

[CR22] Heide EC, Bindila L, Post JM (2018). Prophylactic palmitoylethanolamide prolongs survival and decreases detrimental inflammation in aged mice with bacterial meningitis. Front Immunol.

[CR23] Helyes Z, Nemeth J, Than M (2003). Inhibitory effect of anandamide on resiniferatoxin induced sensory neuropeptide release in vivo and neuropathic hyperalgesia in the rat. Life Sci.

[CR24] Ho WS, Barrett DA, Randall MD. “Entourage” effects of N-palmitoylethanolamide and N-oleoylethanolamide on vasorelaxation to anandamide occur through TRPV1 receptors. Br J Pharmacol. 2008;155:837–46. 10.1038/bjp.2008.324 PMC2597234 18695637

[CR25] Irwin MR, Witarama T, Caudill M (2015). Sleep loss activates cellular inflammation and signal transducer and activator of transcription (STAT) family proteins in humans. Brain Behav Immun.

[CR26] Irwin MR, Olmstead R, Carroll JE (2016). Sleep disturbance, sleep duration, and inflammation: a systematic review and meta-analysis of cohort studies and experimental sleep deprivation. Biol Psychiatry.

[CR27] Keppel Hesselink J, Hekker TAM (2012). Therapeutic utility of palmitoylethanolamide in the treatment of neuropathic pain associated with various pathological conditions: a case series. J Pain Res.

[CR28] Keppel Hesselink J, de Boer T, Witkamp R (2013). Palmitoylethanolamide: a natural body-own anti-inflammatory agent, effective and safe against influenza and common cold. Int J Inflamm.

[CR29] Kesner AJ, Lovinger DM (2020). Cannabinoids, endocannabinoids and sleep. Front Mol Neurosci.

[CR30] Krueger JM (2008). The role of cytokines in sleep regulation. Curr Pharm Des.

[CR31] LoVerme J, Russo R, La Rana G (2006). Rapid broad-spectrum analgesia through acivaion of peroxisome proliferator-acivated receptor-alpha. J Pharmacol Exp Ther.

[CR32] Luongo L, Guida F, Boccella S (2013). Palmitoylethanolamide reduces formalin-induced neuropathic-like behaviour through spinal glial/microglial phenotypical changes in mice. CNS Neurol Disord Drug Targets.

[CR33] Luongo L, Guida F, Boccella S (2013). Palmitoylethanolamide reduces formalin-induced neuropathic-like behaviour through spinal glial/microglial phenotypical changes in mice. CNS Neurol Disord Drug Targets.

[CR34] Marini I, Bartolucci ML, Bortolotti F, Gatto MR, Bonetti GA (2012). Palmitoylethanolamide versus a nonsteroidal anti-inflammatory drug in the treatment of temporomandibular joint inflammatory pain. J Orofac Pain.

[CR35] MattaceRaso G, Russo R, Calignano A (2014). Palmitoylethanolamide in CNS health and disease. Pharmacol Res.

[CR36] Medic G, Wille M, Hemels M (2017). Short- and long-term health consequences of sleep disruption. Nat Sci Sleep.

[CR37] Miner B, Kryger MH (2017). Sleep in the aging population. Sleep Med Clin.

[CR38] Mullington JM, Simpson NS, Meier-Ewert HK (2010). Sleep loss and inflammation. Best Pract Res Clin Endocrinol Metab.

[CR39] Murillo-Rodriguez E, Poot-Ake A, Carrion-Arias O (2011). The emerging role of the endocannabinoid system in the sleep-wake cycle modulation. Cen Nerv Syst Agents Med Chem.

[CR40] O’Sullivan SE, Kendall DA (2010). Cannabinoid activation of peroxisome proliferator-activated receptors: potential for modulation of inflammatory disease. Immunobiology.

[CR41] Prospero-Garcia O, Amancio-Belmont O, Becerril Melendez AL (2016). Endocannabinoids and sleep. Neurosci Biobehav Rev.

[CR42] Re G, Barbero R, Miolo A (2007). Palmitoylethanolamide, endocannabinoids and related cannabimimetic compounds in protection against tissue inflammation and pain: potential use in companion animals. Vet J.

[CR43] Seol TK, Lee W, Park S (2017). Effect of palmitoylethanolamide on inflammatory and neuropathic pain in rats. Korean J Anesthesiol.

[CR44] Skaper SD, Facci L, Barbierato M (2015). N-Palmitoylethanolamine and neuroinflammation: a novel therapeutic strategy of resolution. Mol Neurobiol.

[CR45] Staffe AT, Bech MW, Clemmensen SLK (2019). Total sleep deprivation increases pain sensitivity, impairs conditioned pain modulation and facilitates temporal summation of pain in healthy participants. PLoS One.

[CR46] Vaughn LK, Denning G, Stuhr KL (2010). Endocannabinoid signalling: has it got rhythm?. Br J Pharmacol.

[CR47] Walker MP, Brakefield T, Seidman J (2003). Sleep and time course of motor skill learning. Learn Mem.

[CR48] Worley (2018). The extraordinary importance of sleep; The detrimental effects of inadequate sleep on health and public safety drive and explosion of sleep research. PT.

[CR49] Zygmunt PM, Petersson J, Andersson DA (1999). Vanilloid receptors on sensory nerves mediate the vasodilator acion of anandamide. Nature.

